# Targeting lineage-specific heterogeneity and hypovascular-fibrotic barriers may enable precision immunotherapy in pituitary neuroendocrine tumors

**DOI:** 10.3389/fimmu.2026.1796402

**Published:** 2026-05-01

**Authors:** Jiayu Song, Lin Li, Liusu Ding, Fulian Yang, Han Mao, Keshen Wang, Qiang Li

**Affiliations:** 1 The Second Clinical Medical College of Lanzhou University, Lanzhou, Gansu, China; 2 Department of Neurosurgery, The Second Hospital of Lanzhou University, Lanzhou, Gansu, China

**Keywords:** hypovascular-fibrotic barrier, lineage-specific heterogeneity, pituitary neuroendocrine tumors, precision immuno-oncology, spatial multi-omics

## Abstract

The clinical management of refractory pituitary neuroendocrine tumors (PitNETs) remains a formidable challenge due to pervasive resistance to traditional therapies. Current therapeutic failures appear closely associated with lineage-specific immune heterogeneity and the unique “hypovascular-fibrotic” physical barrier that hinders drug delivery. This review critically examines the biological determinants of therapeutic resistance in the tumor microenvironment (TME) to propose a comprehensive framework for precision immuno-oncology. We highlight how immune infiltration profiles and checkpoint expressions are strongly influenced by transcriptional lineages across PIT1-, TPIT-, and SF1-lineage tumors. We elucidate the “hypovascular-fibrotic” barrier, where low microvessel density and dense collagen deposition restrict therapeutic penetration. We also evaluate advanced strategies, including spatial multi-omics for precise patient stratification and focused ultrasound (FUS) for enhanced drug delivery. In the end, we outline potential combinatorial strategies integrating vascular normalization and metabolic reprogramming to synergize with immune checkpoint inhibitors. This mechanism-driven paradigm shift from empirical treatment to precision immuno-oncology may provide critical insights and novel therapeutic strategies for intractable PitNETs.

## 1 Introduction

As the most prevalent adult sellar tumors and the second most frequent primary central nervous system neoplasm, pituitary neuroendocrine tumors (PitNETs) account for 17.2 percent of cases (1). 2022 WHO classification has rebranded the diagnosis of adenoma as PitNETs (2). Such an important revision reoriented diagnostic orientation to a cell lineage rather than hormone secretion, which is characterized by particular transcription factors: PIT1, TPIT and SF1. Despite their indolent nature in general, there are certain specific subgroups, namely, immature PIT1-lineage tumors and silent corticotropinomas that do exhibit local aggressiveness that essentially has its roots due to lineage of the tumor cells and increased proliferative indices (3).

Although endoscopic transsphenoidal surgery has been shown to successfully treat well-defined microadenomas, it is impossible to perform gross total resection when either a cavernous sinus extension or macroscopic invasion of the sphenoid is present (4, 5). Under these circumstances, any remaining abnormal neoplastic tissue remains in the parasellar space, which serves as a recognized source of early recurrence and development of the disease (6). The dopamine agonist (DAs) is the main drug of choice to treat prolactinomas, but this can be constrained by drug resistance, intolerance, and problems with the use of medications over a long period of time (7). Moreover, radiotherapy has a stable control but at a cost of the risk of delayed collateral hypopituitarism (8–10), and conventional chemotherapy gives quite modest results in refractory disease (11). New therapies are urgently needed. The pathophysiology of PitNETs is not solely due to intrinsic genetic alterations, but also complicated neuroimmune reactions (12). Since the pituitary acts as a unique intersection of neural and endocrine signals, local neurotransmitters and hormone levels significantly affect the behavior of immune subpopulations including T cells and macrophages. This leads to the creation of a dynamic immunosuppressive tumor microenvironment (TME), which ultimately eliminates the effectiveness of conventional immunotherapies (13–17).

In this review, the author will discuss neuro-endocrine circuit mechanisms of immune evasion across PIT1, TPIT, and SF1 lineages in a systematic manner. Our suggestion is that combining spatial multi-omics to obtain an accurate stratification of patients with innovative drug delivery systems will overcome both these physical and biological barriers. Finally, such localised immunosuppression breakdown could reveal important, feasible opportunities to those who fight against refractory PitNETs.

### 1.1 Literature search strategy and selection criteria

We conducted an organized literature search on PubMed. The search included literature that was published since database creation until March 2026. The search strategy implemented a set of Medical Subject Headings (MeSH) and free-text keywords such as: (“Pituitary Neuroendocrine Tumors” OR “PitNETs” OR “pituitary adenoma”) AND (“tumor microenvironment” OR “immunotherapy” OR “immune checkpoint inhibitors” OR “macrophage polarization” OR “hypovascular” OR “spatial multi-omics”).

In order to reduce the selection bias, strict inclusion and exclusion criteria were set. The inclusion criteria included (1): peer-reviewed original research articles, rigorous preclinical models, and clinical trials that directly assessed the neuro-immune axis, barrier mechanisms or immunotherapeutic interventions in PitNETs (2); studies involving spatial multi-omics and single-cell sequencing reporting lineage-specific heterogeneity; and (3) articles in English. The exclusion criteria consisted of (1): unpublished preprints, conference abstracts with no access to the full text and non-peer-reviewed commentaries (2); articles which have not made explicit connection to the tumour microenvironment on a local basis or specific drug delivery systems; and (3) single cases or case series, unless they contained very new findings regarding the response to an immune checkpoint inhibitor or targeted treatment. References of included articles as well as important clinical guidelines were also manually compared to identify further relevant studies.

## 2 The unique immune microenvironment of PitNETs

### 2.1 The cellular landscape of the PitNETs microenvironment

All elements of the TME include immune cells, soluble factors, cytokines, vessels, fibroblasts, and extracellular matrix (ECM), which create a complex intercellular framework that is critical in the onset and development of PitNETs (18). Macrophages and T lymphocytes typically dominate this niche (19), however, they can be characterized by significant functional duality. As an illustrative example, infiltrating tumors by the means of tumor-infiltrating lymphocytes (TILs) could cause a direct antitumor response (cytotoxicity) (20), or vice versa, induce immunosuppression (21). Other cell populations, including B cells, T regs, neutrophils, NK cells, and mast cells, are also present but typically in lower numbers.

Recent transcriptomic profiling reveals that this immune landscape shifts dramatically depending on the specific tumor lineage (22). To give an example, in the PIT1-lineage growth hormone secreting tumors, the immune infiltrate contains mostly CD8 + T lymphocytes and M2 macrophages (23).

### 2.2 Therapeutic targeting of neurotransmitter and hormone receptors in PitNETs

The neuroendocrine system has the pituitary gland as its main organ, and tumor cells are found there, which tends to over express receptors to different neurotransmitters and neuropeptides (24). Dopamine and somatostatin receptors have been examined the most extensively. DRD2 dopamine D2 receptor is the main treatment target of DAs in prolactinoma. Nevertheless, many patients have different levels of dopamine resistance and this condition directly influences the therapeutic effect of DAs. New reports have indicated that an aberrant cholesterol metabolism may disrupt the membrane localization of DRD2 by promoting the development of stress granules, which might be a critical mechanism of dopamine resistance in prolactinoma (25). As a solution, valproic acid (VPA), which is a histone deacetylase inhibitor, has proved to be quite promising. Increasing histone acetylation of Drd2 promoter, VPA increases the transcription of DRD2 and consequently the inhibition of tumor growth through the mTOR-Pttg1 signaling axis. Moreover, the combination of VPA and conventional DAs, like bromocriptine or cabergoline, synergistically reduces PitNET cell proliferation, suggesting an attractive clinical plan to patients who are resistant to DAs (26).

Also, somatostatin receptors (SSTRs), especially isoform 2 and 5, are used as the important points of the treatment (27). Its endogenous ligand, somatostatin (SST), has anti-proliferative, anti-secretory and pro-apoptotic activities. The level of expression of SSTR2 varies greatly between different types of PitNETs; it is most highly expressed in TSH-secreting tumors and growth hormone-secreting tumor, less so in non-functioning gonadotroph PitNETs and corticotroph PitNETs, and least in prolactin-secreting PitNETs and null-cell PitNETs (28). Corticotropin-secreting PitNETs have high levels of SSTR5 expression indicating that SSTR5 could be an important therapeutic target in Cushing’s disease. The drugs that act on specific SSTRs include: Somatostatin receptor ligands (SRLs) such as pasireotide and octreotide. The binding affinity to SSTR5 is very high with pasireotide, whilst octreotide has a higher binding affinity to SSTR2. PasiLAR, or long-acting release of pasireotide, has proven effective in particular under conditions of clinical care of acromegaly as a first-line therapy of biochemical control. Nevertheless, it causes complicated metabolic effects, namely, it could raise the concentration of high-density lipoprotein cholesterol (HDL-C) in a positive manner, but with much higher chances of incident diabetes mellitus due to the strong suppression of insulin and incretin release (29). These unwanted events, such as more extensive gastrointestinal as well as glycemic disorders, demonstrate the partial lack of selectivity of the available SRLs (30).

The regulation of lymphocyte activation pathway controls the level of infiltration by immune cells, especially in pituitary tumors secreting growth hormone wherein aberrant activation of immune cells are driven via mutations in GNAS and this results in different spatial phenotypes (31). At the same time, the acromegaly microenvironment is associated with extensive metabolic toxicity. GH-driven lipolysis and insulin resistance provide a rich environment of lipids and hypertriglyceridaemia, which disrupts cytotoxic T-cell activity and induces the polarization of M2 macrophages. Moreover, the use of multireceptor ligands, including pasireotide, which acts on SSTR-5 to alleviate the primary resistance, leads to unintended worsening of local hyperglycemia and affects glucose equilibrium (29). Such a complicated, two-way interplay among genetic mutations of the tumor and the hormonal excess, followed by the development of metabolic aggressiveness makes a very strong and immunosuppressive TME that reduces the effectiveness of immunotherapeutic treatments.

In spite of the fact that the most recent treatments mainly act through their antiproliferative properties, the presence of resistance mechanisms, including that of dopamine resistant prolactinomas, underscores the weaknesses of focusing merely on these receptors. The widespread distribution of these receptors on matrices and immune cells suggests that traditional neuroendocrine agents have unmet immunomodulatory activity.

### 2.3 Mechanisms of immunosuppression

Neuroimmune crosstalk in PitNETs positively influences the immunosuppressive microenvironment. Remarkably, dopamine signaling is bidirectional. There is a functional tumor-intrinsic D2-receptor-cAMP-axis that preserves cell differentiation indirectly promoting immunogenicity and its malfunction leads to resistance to DAs and the development of local-immune-tolerance (32). Dopamine may directly affect tumor-infiltrating T cells as follows: D1 signaling dampens their action, but D2 signaling decreases the PD-1 level (33, 34).

In addition to dopamine, GABA which is largely produced by TME-inhabiting B cells also emphasizes these regulatory pathways. It blunts the CD8+ T cell cytotoxicity directly, and influences monocytes in such a way that they are directed into IL-10-expressing macrophages (35). Also, GABA also limits the infiltration of T cells by β-catenin activation (36). Other signals play as well, including norepinephrine, which decreases CD8+ T cell efficacy and increases PD-1 expression through β2-ARs, and acetylcholine, which inhibits macrophage inflammation via action on nAChRs (37).

Thus, our hypothesis is that the microenvironment in PitNET is not a static barrier but is rather maintained dynamically by this neuro-immune axis. As tumor and immune cells are mutually regulating the dynamics of neurotransmitters, the key nodes may be therapeutically interrupted by using such combinations, as DAs with GABA antagonists or β-blockers, effectively breaking down the cycle.

### 2.4 Unique anatomical interfaces and the “hypovascular-fibrotic” barrier to drug delivery

The tumor of pituitary is located in a particular vascular niche dominated by the hypothalamic-pituitary-portal system. Contrary to the hard blood-brain barrier (BBB) in other parts of the central nervous system, there is local vessel here that is uniquely fenestrated, which logically determines the exchange of humoral factors and the release of targeted drugs (38).

**Figure 1:**
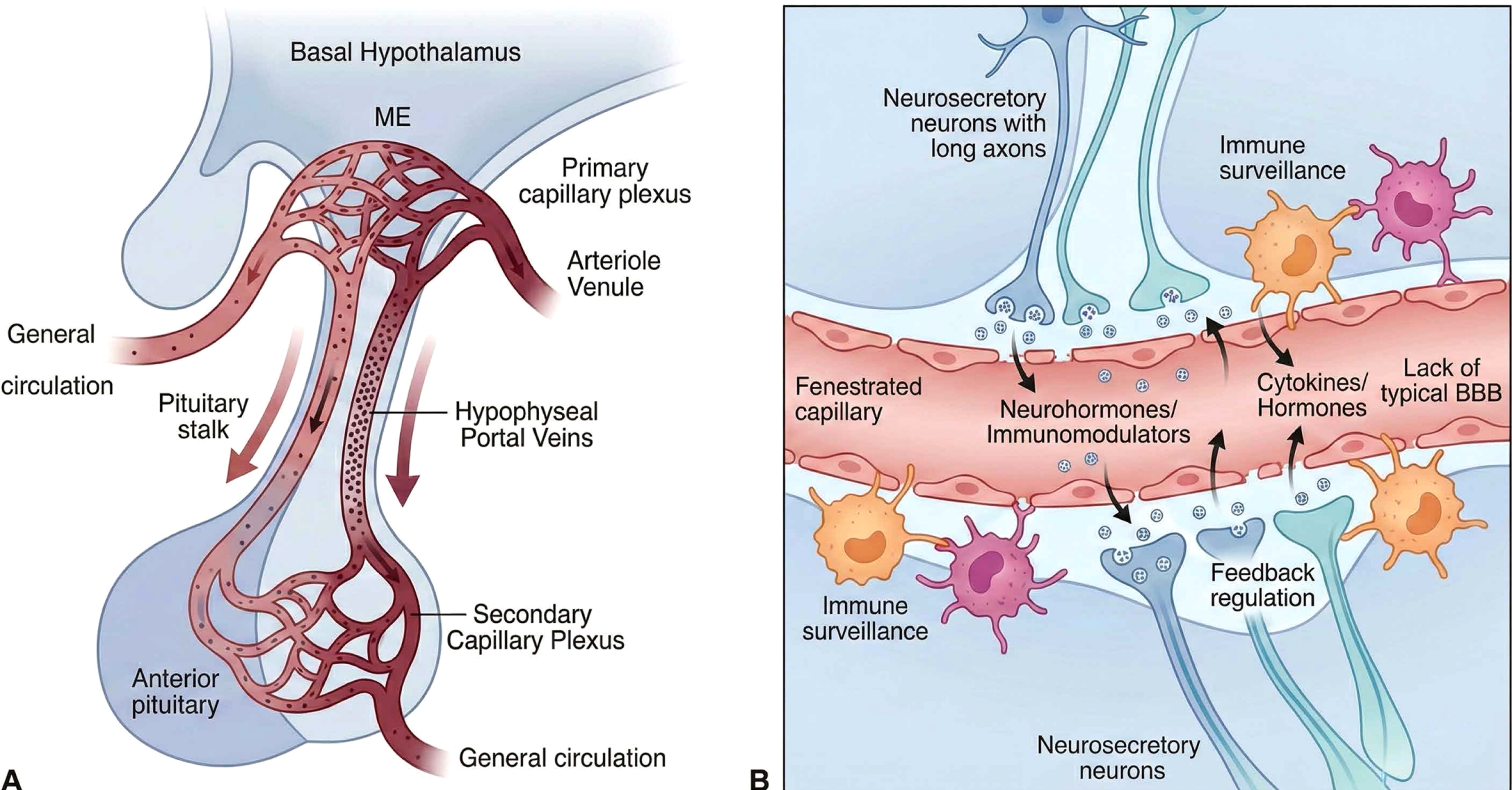
Organization of the hypothalamic-pituitary portal system. **(A)** The portal system connects the median eminence (ME) to the anterior pituitary, allowing direct transport of factors without systemic dilution. **(B)** Fenestrated capillaries at the neuro-vascular interface. The absence of a strict BBB allows for the exchange of hormones and cytokines, supporting feedback regulation and immune surveillance.

The portal system does not enter the systemic circulation but instead allows bypassing it so that high local concentrations of important signaling molecules can be maintained, such as possible immunomodulators (39, 40). Moreover, in contrast to regular brain parenchyma shielded by the BBB, periventricular organs (PVOs) such as the median eminence do not have a usual barrier. Permeability of their fenestrated capillaries enables hormones to easily pass through the blood or cerebrospinal fluid (41). In turn, they become direct physical boundaries of the immune surveillance and neuro-immune communication, which continuously monitors a local humoral condition (42). (**Figure 1**).

Rather than fighting the BBB, therapeutics encounter another specific hypovascular-fibrotic microenvironment. Remarkably, in spite of being of endocrine etiology, PitNETs display very low microscopic vessel density as compared to the normal, and highly vascularized pituitary gland (43) that naturally constrains early drug perfusion levels. This vascular system is not static. In prolactin-secreting PitNETs and non-functioning gonadotroph PitNETs, the proliferation index of stromal endothelial cells is significantly elevated. Although this abnormal angiogenesis driven by factors such as HSPB1 increases the microvascular area, the newly formed blood vessels are often immature and highly permeable, which further exacerbates the hypoxia and poor perfusion within the tumor (44).

Firmer PitNETs also have a significant amount of extracellular type I and III collagens deposited, which further limits this restricted blood flow (45, 46). The ECM is central to this physical barrier. The normal anterior pituitary relies on a fine reticulin framework composed of cross-linked type III collagen to maintain tissue structure. However, in PitNETs, this normal framework is significantly lost and disrupted (47). Instead, a proliferative stroma dominated by excessively deposited fibrous collagen is present (48). This alteration of the matrix not only affects the texture of the tumor but also involves significant changes in the macromolecular composition of the ECM. For example, vitronectin, which is absent in the normal pituitary gland, appears in the tumor microenvironment, while the content of laminin, which has an inhibitory effect on tumor cell proliferation, shows a decreasing trend (49). Single-cell RNA sequencing revealed that tumour-associated fibroblasts (TAFs) exhibited a high degree of functional differentiation, mainly including: myofibroblastic TAFs (mTAFs) primarily driven by the TGF-β signaling pathway, responsible for secreting ECM components and increasing tissue stiffness; inflammatory TAFs (iTAFs) responsible for secreting CCL2, CXCL12, and IL-6 to recruit immune cells; and antigen-presenting TAFs (apTAFs) that highly express MHC class I/II molecules. IFN-γ signaling can effectively inhibit the pro-fibrotic phenotype of mTAF through the STAT3 signaling axis and downregulate its N-cadherin expression, thereby weakening the pro-tumor function of TAF (48, 50). Furthermore, the presence of transitional cancer-associated fibroblasts (CAFs) exhibiting endothelial-mesenchymal transition characteristics in the matrix further increases the complexity of targeted mesenchymal therapy (51). This dense matrix structure mediated by TAFs forms a dual physical and biological blockade against drug penetration and immune cell infiltration. At the physical level, the dense collagen network acts as a barrier, not only limiting the diffusion of oxygen and nutrients, leading to core hypoxia, but also directly confining immune surveillance cells such as T cells within the matrix layer, preventing them from infiltrating the tumor parenchyma to exert their cytotoxic effects (48). At the biological level, spatial transcriptomics revealed strong spatial co-localization between CAFs and SPP1+ tumor-associated macrophages (TAMs) at the periphery of invasive tumors. These two cells synergistically promote matrix remodeling through the SPP1-ITGAV/ITGB1 receptor-ligand pathway. Simultaneously, fibronectin 1 (FN1) and collagen secreted by CAFs directly induce tumor epithelial-mesenchymal transition (EMT) and invasive behavior by binding to integrin receptors on the tumor cell surface (50, 51). Regarding drug resistance, this matrix remodeling barrier is a direct cause of treatment failure. In prolactinomas resistant to dopamine receptor agonists, the degree of type I and type III collagen-mediated fibrosis is significantly higher than in drug-sensitive tumors (52). This matrix-induced drug resistance is primarily achieved through aberrant activation of the TGF-β1/Smad3 signaling pathway in fibroblasts. Further research has shown that the expression of the microRNA miR-93-5p is significantly upregulated in drug-resistant tumors. This upregulation forms a positive feedback loop by targeting Smad7, a natural inhibitor of the TGF-β1/Smad3 pathway, thereby amplifying the fibrotic response and weakening the sensitivity of tumor cells to drugs (53). Blocking this signaling pathway with specific inhibitors has been shown to reverse collagen secretion in fibroblasts, thereby restoring drug sensitivity in drug-resistant cells (52). The composition and physical characteristics of this stromal barrier exhibit significant heterogeneity across different transcriptional lineages and aggressive subtypes; these differences not only shape the landscape of the immune microenvironment but also directly influence the difficulty of surgical resection and drug accessibility. Specifically, the PIT1 lineage—particularly TSH-secreting PitNETs—is characterized by extremely abundant, dense collagen deposition and pronounced features of EMT, resulting in tumors with an exceptionally hard consistency and a highly pro-invasive microenvironment (51, 54, 55). In contrast, tumors of the SF1 lineage exhibit relatively lower overall levels of fibrosis but are enriched with aberrant angiogenesis; while the physical resistance of their stromal barrier is comparatively low, this feature predisposes them to local edema and microinvasion (44, 48, 54). Furthermore, the TPIT lineage restricts the penetration of macromolecular drugs through extensive intratumoral collagen deposition, whereas drug-resistant prolactinomas epigenetically hyperactivate the TGF-β1/Smad3 pathway, triggering severe stromal sclerosis that directly constitutes a core mechanism of drug resistance (52, 53, 56).

Thus, the therapeutic resistance in PitNETs is probably not due to the pressure gradients of convection but the joint effect of inadequate vascularization and the steric hindrance presented by a rigid matrix containing high amounts of collagen.

The latest findings in glioma studies imply that there is an urgent need to move away the concept of passive diffusion towards active penetration. Focused ultrasound (FUS) as one local barrier opening (57) utility has been proven, but more recently, researchers have proposed a magneto-acoustic coupling-based (FUEM) system. Through use of external magnetic fields, this technology can physically pull the nanomedicines, allowing them to actively drill through dense tumor stroma instead of being immobilized by the ECM (58). The given kind of active transport appears to be a promising engineering bypass mechanism when dealing with collagen-caused diffusion limits that occur in PitNETs. Following these developments, we suggest that the translation of active, physically motivated systems to pituitary tumors is a critical next level (59). External physical forces can potentially force open delivery channels even in the face of hypovascularity, and therefore circumvent the fibrotic stroma to overcome the existing bottleneck in the delivery systems.

Although the aforementioned techniques have been demonstrated to bypass the dense ECM found in other experimental solid tumors, their direct applicability to the unique, poorly vascularized fibro-barrier characteristic of PitNETs remains largely speculative. Nevertheless, this represents a biologically plausible engineering hypothesis that warrants rigorous preclinical testing as a means to overcome current diffusion barriers.

### 2.5 Immune checkpoint and inflammatory pathways under neural modulation

In PitNETs, the immune milieu has a very high degree of dynamism due to the genetic nature of the tumours and the neuroendocrine lineages. We can observe this through expression of the programmed death-ligand-1 (PD-L1) protein. Instead of being found randomly, PD-L1 distribution is tightly regulated based on the underlying transcriptional history of the cell. On the other hand, PD-L1 expression is usually high in PIT1-lineage tumors whereas it is much smaller in SF1 and TPIT lineages (19, 60, 61). To support this, a mega cohort study on the relationship between PD-L1 and GH secretion was conducted by Cossu et al., who found high correlations between elevated PD-L1 and GH secretion, and low with FSH/LH. Surprisingly, the expression of this checkpoint correlates directly with the aggressiveness of the tumor. According to the Trouillas grading system, in case of the “proliferative” tumors, there is significantly higher probability of PD-L1 overexpression with frequent concomitant p53 accumulation (61). Based on these trends, it seems that PIT1-lineage cells actively increase inhibitory checkpoints as an avoidance mechanism to the immune surveillance and speed up their own development (60).

Regardless of lineage, any mutations in specific genes will drastically alter the local microenvironment through commandeering inflammatory pathways. As an example, in somatotroph PitNETs, AIP gene mutations cause strong local inflammation. The mutated cells are upregulating CCL5 synthesis which activates CCL5/CCR5 pathway. Not only does it draw the macrophages into the tumor bed but also directs them towards an immunosuppressive M2 state. Concurrently, such STAT3 phosphorylated cells have an effect of increasing the production of pro-inflammatory cytokines, such as IL-6, that promote a more aggressive clinical phenotype. Likewise, corticotrophomas that have distinct genetic alterations have a markedly different landscape of immunity than wild-type cases and commonly have heavy infiltrates of immature CD4 + and CD8 + T cells (19).

The pituitary tumour cell populations produce both soluble factors that induce angiogenesis and recruit macrophages and neutrophils into the local niche (19, 62). CCL2, CXCL8, and VEGF-A are produced by pituitary tumour cells and TAFs. Upon arrival, the tumor-derived metabolites with the presence of IL-4 and GM-CSF push these macrophages to a pro-tumorigenic M2 phenotype. In turn, the said M2 macrophages release CCL17, which directly affects the tumor cells, through the CCR4/mTORC1 signaling cascade, driving the process of EMT and further invasion (19).

**Figure 2:**
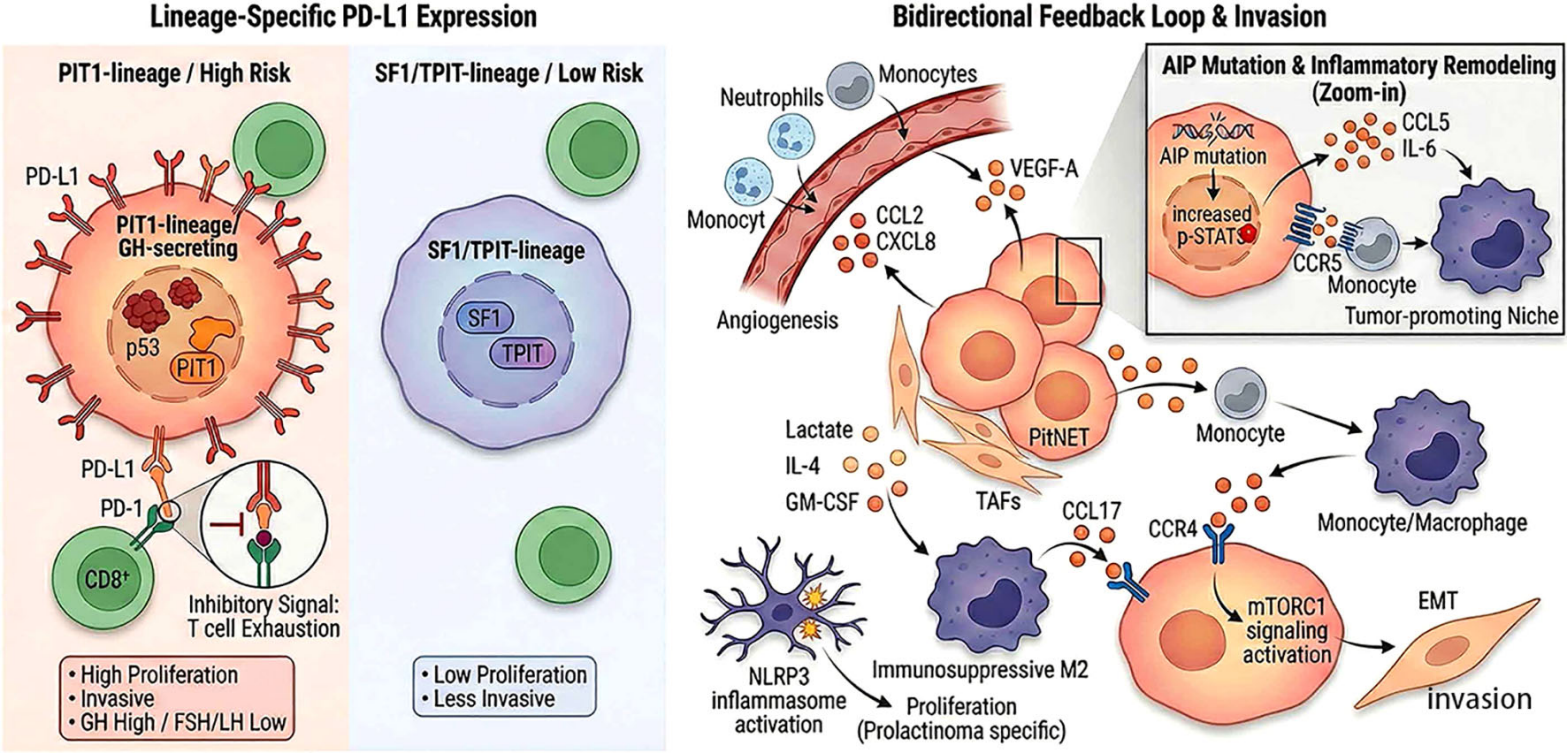
The landscape of immune microenvironment in PitNETs: lineage-dependent checkpoints and inflammatory feedback loops. This schematic illustrates the lineage-specific immune mechanisms in PitNETs. Left: PIT1-lineage tumors exhibit elevated PD-L1 expression and CD8+ T cell exhaustion compared to SF1/TPIT subtypes. Right: A bidirectional feedback loop drives tumor progression, where tumor-educated M2 macrophages secrete CCL17 to activate the tumor CCR4-mTORC1 axis, promoting EMT and invasion. Additionally, AIP mutations amplify this inflammatory niche via p-STAT3-mediated CCL5/IL-6 secretion to recruit monocytes.

Finally, the immune evasion of PitNETs depends on a complicated interplay between the lineage identity, the mutational profile and the controlled inflammatory cycles. Simplifying our view about certain pathways such as CCL5/CCR5 and CCL17/CCR4 axes will offer a good theoretical basis to tailor upcoming immunotherapy to different molecular subtypes (**Figure 2**).

## 3 Immunotherapy strategies for PitNETs

### 3.1 Immune checkpoint inhibitors

Immune checkpoint inhibitors (ICIs) are able to inhibit the pathways such as PD-1/PD-L1 to re-activate the exhausted T cells, which is the most rapidly translated approach in the clinics (19). Systematic reviews show that anti-PD-1/PD-L1 treatments provide approximately 30% objective response rates in patients with refractory PitNETs and pituitary carcinoma (63). However, existing relevant literature remains limited in scope and characterized by significant heterogeneity regarding patient selection criteria, tumor subtype classification, and study design protocols. Although these preliminary clinical findings demonstrate promising prospects for a class of diseases that have historically proven difficult to effectively control through pharmacotherapy, definitively establishing a significant survival benefit still necessitates rigorous and comprehensive validation through large-scale prospective clinical trials.

This effectiveness has been proven in real-life settings. Coincidentally, greater tumoral PD-L1 expression seems to suggest more favorable outcomes, providing a good basis on which future patient stratification can be performed based on biomarkers (64). Nevertheless, even with these developments there are still notable challenges of varying responses to treatment and safety concerns. According to the available pharmacovigilance data, they may cause particular endocrine toxicity, including hypopituitarism, whose onset follows different time courses. To counterbalance these clinical benefits with adverse reactions finally implies severe surveillance and management guidelines (65).

### 3.2 Targeting myeloid cells

Recently developed methods in single-cell transcriptomics have characterized different subpopulations of macrophages strongly associated with bone invasion and defined some molecular targets of aggressive PitNETs (66). As a result, it is likely that the future will bring a change in therapeutic strategies away from macrophage depletion broadly towards more specific phenotypic reprogramming. Conversely, it is implied by research that anti-PD-L1 antibodies may signal through signaling cascades like EGR1 and HSP90AB1 to cause indirect macrophage repolarization, highlighting the possible synergy between ICIs and myeloid-targeted treatments (67). However, more sophisticated synthetic biology tools are being applied in the design of chimeric cytokine receptors. The receptors make macrophages capable of transducing inhibitory microenvironmental signals, including IL-10 or TGF-β, into pro-inflammatory, anti-tumor signals, changing immunosuppressive signals into immunostimulatory signals (68).

### 3.3 Targeted delivery technologies

With regard to biochemical delivery, lipid nanoparticles, in particular the controlled regulation of the lengths of their PEG-lipid anchor chains, have great potential in improving the intracerebral delivery of nucleic acid therapeutics such as mRNA vaccines and siRNA. It creates a multifunctional intervention platform based on genes (69). Besides, they are also multifunctional carriers with very high potential of the dendritic trans-BBB delivery of immunomodulators (70). FUS and microbubble technology has since become less of a means of transient barrier disruption in the context of physical interventions and more of an integrated theranostic platform. With an accompanying use of ultrasound guided flow imaging, it can be used to measure and regulate brain circulation during procedures, thus, significantly increasing safety (71). Moreover, the new ultrasound systems that combine remote magnetic control allow not only controlling opening of BBB, but also modulating the immune microenvironment in real time. This permits a two-way approach to PitNETs treatment, allowing combining the targeted drug administration with microenvironmental regulation (72).

## 4 Translational challenges in neuroimmunoregulation for PitNETs treatment

### 4.1 Diagnostic challenges in the era of immunotherapy

The intrinsic immunosuppressive microenvironment of PitNETs represents a major barrier to the effectiveness of immunotherapy. Single-cell and spatial transcriptomics have demonstrated considerable heterogeneity of both neoplastic components and the composition as well as spatial organization of immune infiltrates (22). Moreover, this immunosuppressive condition is maintained through a complicated web containing macrophages, T cells and dendritic cells (73). Therefore, in this highly heterogeneous immune suppressive setting, organized by multidimensional signaling circuits, the anti-tumor activity of monotherapy using one type of immunotherapy is substantially reduced.

MRI-detected pituitary sellar lesion growth under the treatment of ICIs in PitNETs is a common clinical conundrum. The enlargement may be actual tumor progression (TTP), pseudo-progression induced by the immune system reaction, or immune-mediated hypophysitis (IH). It is important to differentiate between these entities as pseudoprogression or IH is often interpreted as TTP which may cause termination of otherwise effective immunotherapy prematurely (74).

It complicates identifying a case of IH because initial systemic signs- including fatigue and headache- are similar to the effects of the tumor mass and cachexia (75). However, it has a specific endocrine pathway that provides a greater indication of the diagnosis.

The traditional imaging guidelines tend to miss such abnormal immune responses (74). The iRANO criteria have thus taken on a fundamental role in the future PitNET trials. iRANO is safe since it provides clinicians with the opportunity to keep their original treatment even when signs of lesion growth are apparent and therefore can help overcome misidentification of pseudoprogression (76). More recent MRI technology also helps make this separation: pseudoprogression typically displays less vascular plasma volume on dynamic contrast scans, while IH has a hallmark of generalized pituitary enlargement and thickening (74).

When this condition is verified, the response to prompt glucocorticoid therapy by IH or ICI-induced AI is very quick, with the symptoms being resolved in most instances (77). With appropriate management of hormones, most people will be able to restart their ICI therapy safely. Finally, these subtle diagnostic processes are essential to gain maximum benefit out of the therapeutic runway of PitNETs.

### 4.2 Limited infiltration and functional exhaustion of immune effector cells

Immunotherapy effectiveness depends on precisely defining the location and long-term operation of effector cells in the tumor environment. Nevertheless, PitNETs have two major obstacles to this stage both physically and functionally. Functionally, regardless of successful T cell infiltration, the PD-L1 upregulated in the TME binds to PD -1 receptors causing the exhaustion of T cells, which is one important mechanism enabling PitNETs to elude the immune response (78). Moreover, the results of spatial transcriptomics in aggressive subtypes suggest that metabolic deregulation in the PI3K-AKT signal transduction pathway might support local immune tolerance additionally (79).

### 4.3 Mechanisms of non-response to immunotherapy

Although ICIs are theoretically promising, clinical reports demonstrate that about 70 percent of aggressive PitNETs fail to respond to therapy (64). This poor success rate is mostly due to the naturally low tumour mutational burden (TMB) of PitNETs (80), which cannot produce the immunogenic neoantigen necessary to effectively activate the T cells (81).

On the tissue plane, TME is actively inhibiting the immunity with the changes of metabolism. Namely, tumor-derived lactate switches macrophages to the protumorigenic M2 status (82). At the same time causing metabolic stress of nearby CD8 + T cells, increasing their exhaustion and apoptosis (83). In an attempt to respond to such stress, T cells tend to increase activity of other co-inhibitory receptors such as TIM-3 and LAG-3. The upregulation forms an excess inhibitory system that can easily circumvent blockade of a single target (84).

After all, such multidimensional interaction between poor antigenicity, target mismatch, metabolic depletion, and structural sheltering is obviously the key explanation behind the exasperating failure rates of aggressive PitNETs undergoing immunotherapy (64).

## 5 Combination therapy strategies

### 5.1 Synergistic mechanisms between immunotherapy and conventional treatments

Traditional therapies such as surgery, radiotherapy, and drug treatment are used to manage the tumor load while they will intrinsically affect the TME, which provides a pivotal opportunity to use immunotherapy. The main treatment of most PitNETs is endoscopic resection, which does not merely provide mechanical cytoreduction. It facilitates reversing systemic immunosuppression due to the rapid decrease in tumor mass, and thus creates space in the adjuvant therapy with immunotherapy (85, 86). Also, surgical tissue damage produces antigens that can elicit *in vivo* vaccination to prime T cells.

Radiation therapy is also combined very effectively with ICIs. In addition to delivering a precise tumor control, stereotactic radiosurgery (SRS) also serves as a powerful immunomodulator (87). Radiations induce immunogenic cell death releasing antigens and damage-associated molecules that effectively turn the TME into an immunological state of being both cold and hot. This immune-cell influx improves local ICI effectiveness and may cause an abscopal effect on distant metastases (88). Considering the effectiveness of other combination treatments in other CNS tumors such as gliomas, there is a high potential in applying the radio-immunotherapy approaches to PitNETs (89).

Although they are primarily used as conventional endocrine suppressors, DAs and somatostatin analogs (SSAs) do exhibit specific immunomodulatory properties, including changing the polarization of macrophages in the stroma (90, 91). These agents are summarized in **Table 1**, which includes direct evidence from PitNETs as well as supportive mechanistic findings from related inflammatory and neurological models. Collectively, these studies suggest that dopaminergic agents may influence diverse immune functions, although much of the non-PitNET evidence remains extrapolative.

**Table 1:** Immunomodulatory effects of dopaminergic agents: direct evidence in PitNETs and supportive findings from related inflammatory and neurological models.

Agent	Target receptor/Cell	Immunomodulatory mechanism	Experimental model	Reference
Cabergoline	DRD2/CD8+ T cells	Promotes cytotoxic CD8+ T cell infiltration and upregulates GrzB/Perforin expression.	Prolactinoma	(108)
Pramipexole	DRD3/Tregs	Enhances Treg immunosuppressive function and IL-10 secretion via TGF-β signaling.	Parkinson’s Disease	(109)
Pramipexole/Rotigotine	DRs/Tregs & Monocytes	Induces anti-inflammatory transcriptional reprogramming in Tregs and monocytes.	Parkinson’s Disease	(110)
Dopamine	DRD5/Macrophages	Promotes M2 polarization and inhibits M1 polarization.	Colitis (Mouse)	(111)
A-68930 (D1 Agonist)	DRD1/Microglia	Promotes autophagy-dependent degradation of NLRP3 inflammasome.	Alzheimer’s Disease (Mouse)	(112)
Dopamine	DRD1/Macrophages	Inhibits NLRP3 inflammasome assembly via cAMP-dependent ubiquitination.	Systemic Inflammation	(113)
Dopamine	Non-receptor/Microglia	Inhibits NF-κB p65 nuclear translocation via formation of dopamine quinone.	Neuroinflammation (Microglia)	(114)
Dopamine	DRD1-5/T cells	Biphasic regulation: activates resting T cells but inhibits activated T cells.	General Immunology	(115)
Dopamine Agonists	Multiple	Comprehensive modulation of innate and adaptive immunity bridging CNS and periphery.	–	(116)
Temozolomide/ICIs	DNA/PD-1	Therapeutic options for dopamine-agonist resistant/aggressive PitNETs.	Refractory Prolactinoma	(117)

### 5.2 Vascular normalization and metabolic reprogramming

In the study of gliomas, the VEGF pathway has been shown not merely inhibit angiogenesis, but also restores abnormal vessel, which relieves hypoxia and allows the infiltration of cytotoxic T lymphocytes (92). Since PitNETs use a distinct type of semi-open portal blood supply (93), it is feasible that anti-angiogenic drugs might cause the same kind of vascular normalization, therefore eliminating the physical obstacles that keep immune cells away from the center of the tumor. However, direct empirical evidence for this mechanism within the PitNET microenvironment remains to be established. In addition to remolding the vessel, there is another important way to overcome immunosuppression by attacking tumor metabolism. Also, immune tolerance of refractory prolactinomas is closely associated with spatial transcriptomics and PI3K-Akt dysregulation (79). These findings, combined with the fact that metabolic manipulation of microglia can improve the effectiveness of immune checkpoint blockade (ICB) in gliomas (94), suggest that a parallel combination of metabolic regulators/ICIs could also change the immuno-metabolic environment of PitNETs. Nevertheless, this remains an inferential strategy that requires targeted *in vivo* validation. Lastly, new FLASH radiotherapy is worth mentioning. It has been proven to obliterate medulloblastoma cells without damaging healthy brain tissues and creating positive conditions in immune system (95), providing an intriguing conceptual framework of testing safer and less toxic combinations of radio-immunotherapy in PitNETs.

### 5.3 Biomarker-guided individualized combination therapies

Due to the extreme molecular heterogeneity of the PitNETs (22, 73), it is evident that the future treatments should be shifted away empirical regimens towards biomarker-based precision medicine. This change can be accomplished with a high-throughput profiling approach, especially single-cell and spatial transcriptomics, as the realistic molecular platform. These improved tools will allow us to individualize combination therapies by identifying distinct immune landscapes in various subtypes, and therefore, apply them specifically to the TME.It is thus recommended that the therapy paradigm be stratified, with the major biomarkers being the expression of PD-L1, PI3K-AKT pathway activity, and alternative splicing variants (78, 79, 96). Conversely, to address the issue of treatment resistance in refractory prolactinomas that have been promoted due to the presence of high levels of lactate metabolism and the PI3K-AKT system the combination of ICIs with metabolic inhibitors appears as an extremely logical strategy.

## 6 Future directions and translational prospects

### 6.1 The triad of innovative immunostrategies, intelligent delivery, and precision stratification

Bioengineered methods such as adoptive cell therapies are becoming popular due to the limitations of traditional immune checkpoint inhibitors. As another example, NK cells also exhibit non-MHC-restricted cytotoxicity and have a beneficial safety profile (97). At the same time, identification of shared tumor antigens is a key component of the next-generation of cancer vaccines (98). New systems based on either using M13 bacteriophage nanoparticle with STING agonists have now provided dependable systems to evoke long-lasting immune memory to neoantigens (99, 100). There are additional opportunities offered by antibody engineering: bispecific tetravalent antibodies that induce selective apoptosis and do not cause hepatotoxicity (101) and nanobody linkers targeting both CTLA-4 and PD-L1 increase tissue penetration and decrease systemic toxicity over traditional blockades (102).

Biochemically, biomimetic nanotechnologies increase BBB permeability and cause immunogenic cell death, making them a good link between drug delivery and immune stimulation (103). Implantable biodegradable scaffolds enable locally-sustained release of immunomodulators to address residual postoperative lesions. This method will assist in the removal of micrometastases at minimal systemic adverse effects compared to conventional treatment (104).

To finally achieve this goal, such targeted therapies need to be matched with appropriate patients based on high-dimensional biomarkers. Even more detailed molecular subtyping is possible by studying the diversity of transcriptomic splicing rather than the typical gene expression (96). Finally, the incorporation of these multi-dimensional elements into predictive models will become an unavoidable part of an efficient patient stratification in future.

### 6.2 Integrated decision systems for spatial multi-omics and radiomics

On microscopic scale, a combination of single-cell RNA sequencing and spatial transcriptomics precisely outlines complex *in vivo* cell networks (105, 106). In much the same way that this spatial multi-omics approach has managed to characterize glioma margins, we anticipate it could help identify the micro-anatomical areas that induce PitNET invasiveness and immune escape (107). Macroscopically, there is a complementary approach to radiomics as a non-invasive technique. The integration of this detailed molecular information with whole-tumor macroscopic imaging generates a powerful radiogenomic structure. The pragmatic application makes it possible to perform in-depth preoperative evaluations of tumor biology and immune environments, and finally establish the basis of personalized treatment of PitNETs.

## 7 Conclusions

Because immune profiles are tightly associated with specific transcriptional lineage, nonspecific immunotherapies may face significant limitations in an unselected population. In addition to that, the “hypovascular-fibrotic” barrier seems to restrict entry of medicines to refractory PitNETs.

To address these challenges, exploring a transition from empirical approaches toward mechanisms-based treatments warrants further investigation. The integration of spatial multi-omics for precise patient stratification, alongside bioengineering techniques such as targeted ultrasound to bypass physical barriers, offers a rational conceptual framework. Although more stringent validation is required prior to routine use in the clinic, shifting towards precision immuno-oncology may represent a promising direction for managing the aggressive and recurring nature of PitNETs.
